# *Pasteurella multocida* filamentous hemagglutinin B1 (*fhaB1*) gene is not involved with avian fowl cholera pathogenesis in turkey poults

**DOI:** 10.1186/s12917-025-04668-1

**Published:** 2025-03-26

**Authors:** Rohana P. Dassanayake, Robert E. Briggs, Bryan S. Kaplan, Harish Menghwar, Carly Kanipe, Eduardo Casas, Fred M. Tatum

**Affiliations:** 1 Ruminant Diseases and Immunology Research Unit, United States Department of Agriculture, Agricultural Research Service, National Animal Disease Center, Ames, IA 50010 USA; 2 Oak Ridge Institute for Science and Education (ORISE), ARS Research Participation Program, Oak Ridge, TN 37830 USA; 3 Infectious Bacterial Diseases Research Unit, United States Department of Agriculture, Agricultural Research Service, National Animal Disease Center, Ames, IA 50010 USA

**Keywords:** *Pasteurella multocida*, Fowl cholera, Filamentous hemagglutinin, *fhaB1*, Mutants

## Abstract

**Background:**

*Pasteurella multocida* is a Gram-negative coccobacillus and is the causative agent of fowl cholera in avian species. *P. multocida* expresses two large filamentous hemagglutinin (FhaB) proteins encoded by *fhaB*1 and *fhaB*2 genes. Previously, it was demonstrated that *P. multocida* FhaB2 is an important virulence factor in the development of fowl cholera disease. In the current study, we examined the potential role of FhaB1 in fowl cholera disease development. An *fhaB1* deletion mutant, devoid of foreign DNA, was constructed using a temperature sensitive plasmid in a well-characterized *P. multocida* avian strain P-1059 (A:3).

**Results:**

Real-time PCR assay confirmed the expression of full-length *fhaB1* mRNA in the wild-type parent strain and truncated *fhaB1* mRNA in the Δ*fhaB1* mutant strain. Both parent and the mutant strain produced biofilm; however, the Δ*fhaB1* mutant produced significantly lower amounts of biofilm. Turkey poults were challenged intranasally and intramuscularly to assess the virulence of the *fhaB1* mutant and the wild-type parent strains. Contrary to our expectation, inactivation of *fhaB1* did not reduce virulence by either challenge route.

**Conclusions:**

These findings indicate that this large and highly conserved FhaB1 protein is not necessary for the development of acute fowl cholera disease in turkeys.

**Supplementary Information:**

The online version contains supplementary material available at 10.1186/s12917-025-04668-1.

## Background

*Pasteurella multocida* is a heterogeneous species and causes respiratory or systemic disease such as fowl cholera in birds, hemorrhagic septicemia in water buffalo and cattle, pneumonic pasteurellosis in ruminants, atrophic rhinitis in swine, and snuffles in rabbits. Based on capsular polysaccharide composition, *P. multocida* is classified into five serogroups, A (hyaluronic acid), B, D (heparosan), E, and F (chondroitin) [1, 2]. Additionally, 16 somatic (or lipopolysaccharide) serovars have been identified based on the Heddleston agar gel diffusion serotyping method [3]. Serogroup A strains, independent of somatic serovar are associated with causing fowl cholera although serovar A:1, A:3, and A:3,4 are most frequently associated with this disease [4]. Fowl cholera is a highly contagious disease that results in considerable economic losses to the poultry industry world-wide [5].

It is thought that *P. multocida* enters tissues of birds through mucosal membranes of the pharynx or upper respiratory tract [6]. Also the bacterium may enter the host through the conjunctiva [7]. Fowl cholera can occur as a peracute/acute syndrome where signs of infection may be present for only hours prior to terminal bacteremia. Signs which often accompany peracute/acute fowl cholera are fever, anorexia, diarrhea, mucous discharge from the mouth, and tachypnea. Turkeys are more susceptible to peracute/acute fowl cholera than chickens and older chickens are more susceptible than are young birds [8, 9]. General signs of chronic fowl cholera are characterized by localized infections of the leg and wing joints, wattles, sinuses, footpads, and sterna bursae. Inflammation of the conjunctiva, pharyngeal lesions, dyspnea, lameness, and torticollis are also known to occur in chronically infected birds [5].

Some reported virulence determinants of *P. multocida* include capsule [10], lipopolysaccharide [11], iron regulated proteins [12], outer membrane proteins OmpA [13], OmpH [14], sialylation of outer membrane components [12, 15], and the putative adhesin, filamentous hemagglutinin B2 (FhaB2) [16]. *P. multocida* expresses two large FhaB proteins encoded by *fhaB1* and *fhaB2* genes. Both proteins exhibit a high degree of similarity with diverse members of the *Pasteurellaceae* [17, 18]. This family of proteins are regarded as important virulence factors that play crucial roles in interactions between these bacteria and host organism. FhaB proteins are both surface associated and secreted to the extracellular milieu by means of two-partner secretions systems involving the transporter FhaC. Interestingly, the FhaB1 and FhaB2 proteins of *P. multocida* are devoid of the integrin-binding RGD motif present in many FhaB cognates. Adherence of pathogens to host cells contributes to colonization, invasion and spread of bacteria into tissues and secondary sites of infection [6, 15]. The large secreted FhaB2 protein of *P. multocida* P-1059 is necessary to establish fowl cholera in turkeys upon mucosal exposure [16]. It is likely that FhaB2 functions as an adherence molecule involved in colonization and/or bacterial invasion of respiratory mucosal surfaces in turkeys. Due to the key role FhaB2 plays in pathogenesis, vaccine trials using recombinant FhaB2 peptides of *P. multocida* strain P-1059 (A:3) were conducted which demonstrated that anti-FhaB2 antibodies effectively protected turkeys against homologous challenge and heterologous challenge with *P. multocida* χ73 (A:1) [19, 20]. The broad cross-protection afforded by this recombinant FhaB2 peptide vaccine is likely attributable to the high degree of conservation of *P. multocida* FhaB2 proteins.

Genomic sequencing has identified another filamentous hemagglutinin gene (*fhaB1*) present in *P. multocida* strain P-1059, and in many other avian strains of *P. multocida* sequenced to date. The open reading frame of P-1059 *fhaB1* is large, 7,854 bp, directly up stream of *fhaB2*, and is highly conserved with the *fhaB1* of other *P. multocida* isolates [21]. The *fhaB1* and *fhaB2* predicted protein products of strain P-1059 are divergent with about 50% identity across the majority of the proteins. In the current report an “unmarked” *P. multocida* P-1059 *ΔfhaB1* deletion mutant was constructed, and the virulence of the mutant was assessed in a turkey model.

## Methods

### Bacterial strains

*Pasteurella multocida* P-1059 (A:3) wild-type [9], filamentous hemagglutinin B2 (Δ*fhaB2*) [16] capsular (Δ*hyaE*) [22], and filamentous hemagglutinin B1 (*ΔfhaB1*) (constructed in this study) mutant strains used in this study were cultured on BD Difco™ dextrose starch agar (BD Biosciences, Franklin Lakes, NJ) supplemented with 5% sheep blood. Freezer stocks were prepared by harvesting bacteria (from blood agar plates) into Columbia broth (BD Difco™) containing 10% glycerol and preparing 1 ml aliquots into cryovials, which were then stored at -80^°^C.

### Recombinant DNA techniques

**Fig. 1:**
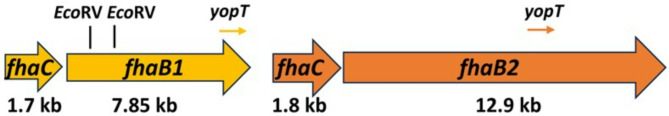
Genetic organization of the filamentous hemagglutinin (*fha *) gene cluster of *Pasteurella multocida* strain P-1059. *yopT* = *Yersinia enterocolitica* outer protein T

**Fig. 2:**
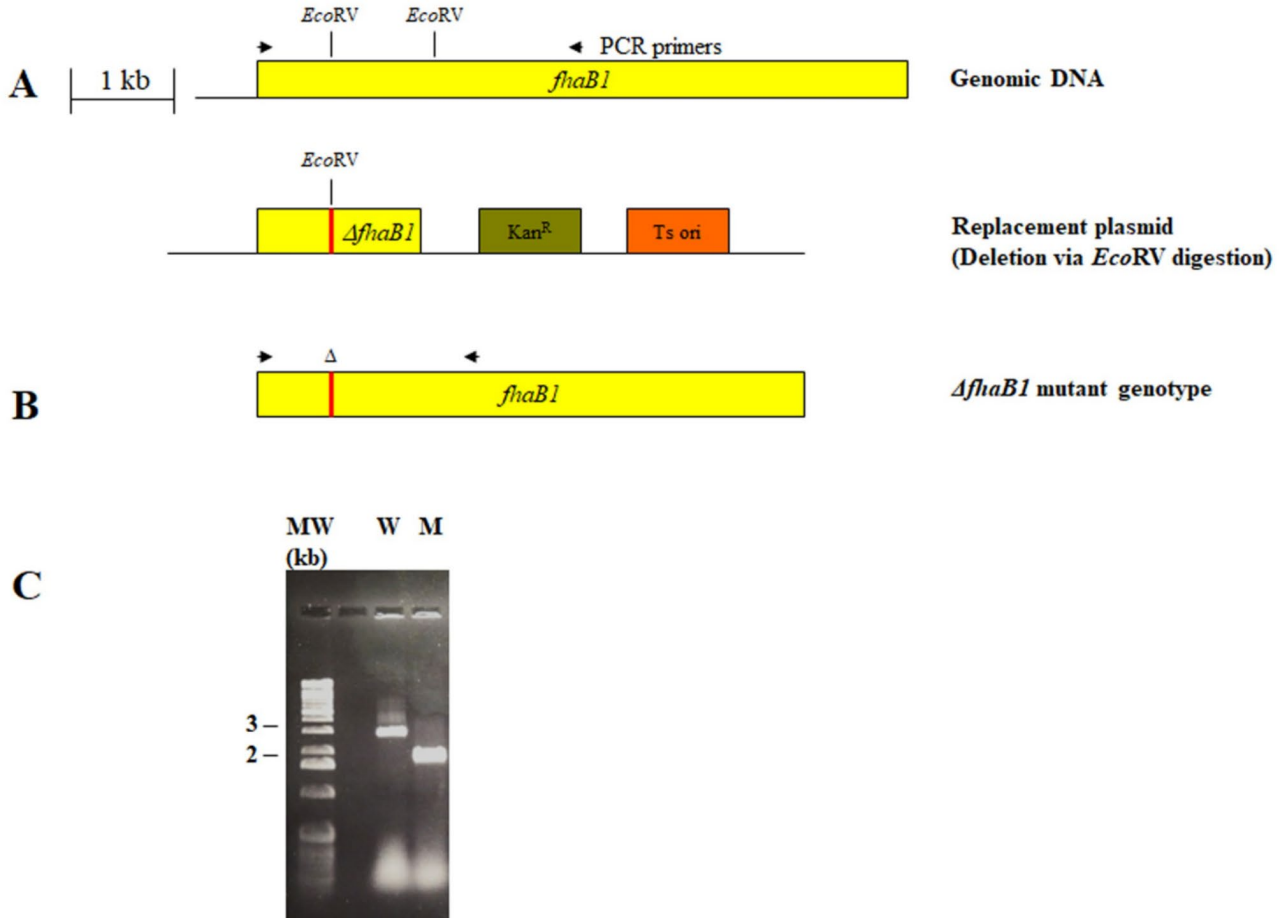
Schematic depiction of *Pasteurella multocida* strain P-1059 filamentous hemagglutinin B1 ( *fhaB1 *) gene. **A**). The arrows indicate the priming sites used to amplify 2,867 bp fragment of *fhaB1* by PCR with the primer pair, fhaB1F/fhaB1R (Table 1). **B**). After digestion with *Eco*RV, 1,090 bp was deleted from *fhaB1*. The deleted fragment was joined to the Ts origin and kanamycin resistance element to create the replacement plasmid used to generate the Δ*fhaB1* deletion. **C**). The wild-type parent (W) and Δ*fhaB1* mutant (M) strains of *P. multocida* were analyzed by PCR using the primer pair fhaB1F/fhaB1R

Genetic organization of *fhaB* and *fhaC* is shown in Fig. 1. A region of the *fhaB1* gene of *P. multocida* P-1059 (Fig. 2A) was amplified by PCR using the primer set fhaB1 forward and fhaB1 reverse (Table 1). All the primers used in this study were custom synthesized by Integrated DNA Technologies, Inc. (Coralville, IA). PCR reactions were performed using the EasyStart PCR mix in a tube as per manufacturer’s description (Molecular BioProducts, San Diego, CA) using *P. multocida* P-1059 cells as template. The PCR reaction produced a product of 2872 bp in length which was inserted into plasmid, pCR2.1 using the TopoTA cloning kit (Invitrogen Life Technologies, Carlsbad, CA) then introduced into electrocompetent *Escherichia coli* Top10 cells. Afterwards, the *E. coli* was spread onto LB agar plates containing ampicillin (100 μg/ml) and grown overnight at 37ºC. Single bacterial colonies were inoculated into LB broth containing ampicillin and grown overnight at 37ºC with shaking. Plasmid DNA was isolated from clones positive for *fhaB1* insert using Spin-preps (Qiagen Inc. Valencia, CA). The *fhaB1* fragment was excised from pCR2.1 by *Eco*R1 digestion and transferred to the *Eco*R1 site of plasmid pBCSK (Stratagene Inc., La Jolla, CA) which had been modified by removing the *Eco*RV recognition site within the multiple cloning site (MCS) to generate plasmid, pBCSKfhaB1. Plasmid, pBCSKfhaB1, was recovered and purified, then subjected to *Eco*RV digestion to create a deletion within *fhaB1*. The deletion site was sealed by treatment with T4 DNA ligase. A Tn903 kanamycin resistance cassette, Kan^R^, (Genblock, Pharmacia Biotech Inc., Piscataway, NJ) was ligated into pBCSKΔfhaB1 at the *Bam*HI site of the MCS to produce plasmid, pBCSKΔfhaB1Kan^R^. The temperature sensitive (Ts) plasmid origin of replication of plasmid, pTsoriCT109GA189, was isolated by digestion with the restriction enzyme *Bss*HII as was plasmid pBCSKΔfhaB1Kan^R^. The two *Bss*HII treated plasmids were combined using T4 ligase. The mixture was electroporated into P-1059, plated onto Columbia blood agar base plates containing kanamycin (25 μg/ml) and incubated at the permissive temperature for replication. Only plasmid containing both the Ts origin-of-replication together with the MCS of pBCSK possessing the kanamycin resistance element with the *fhaB1* fragment were capable of sustaining growth of *P. multocida* under this condition.

**Fig. 3:**
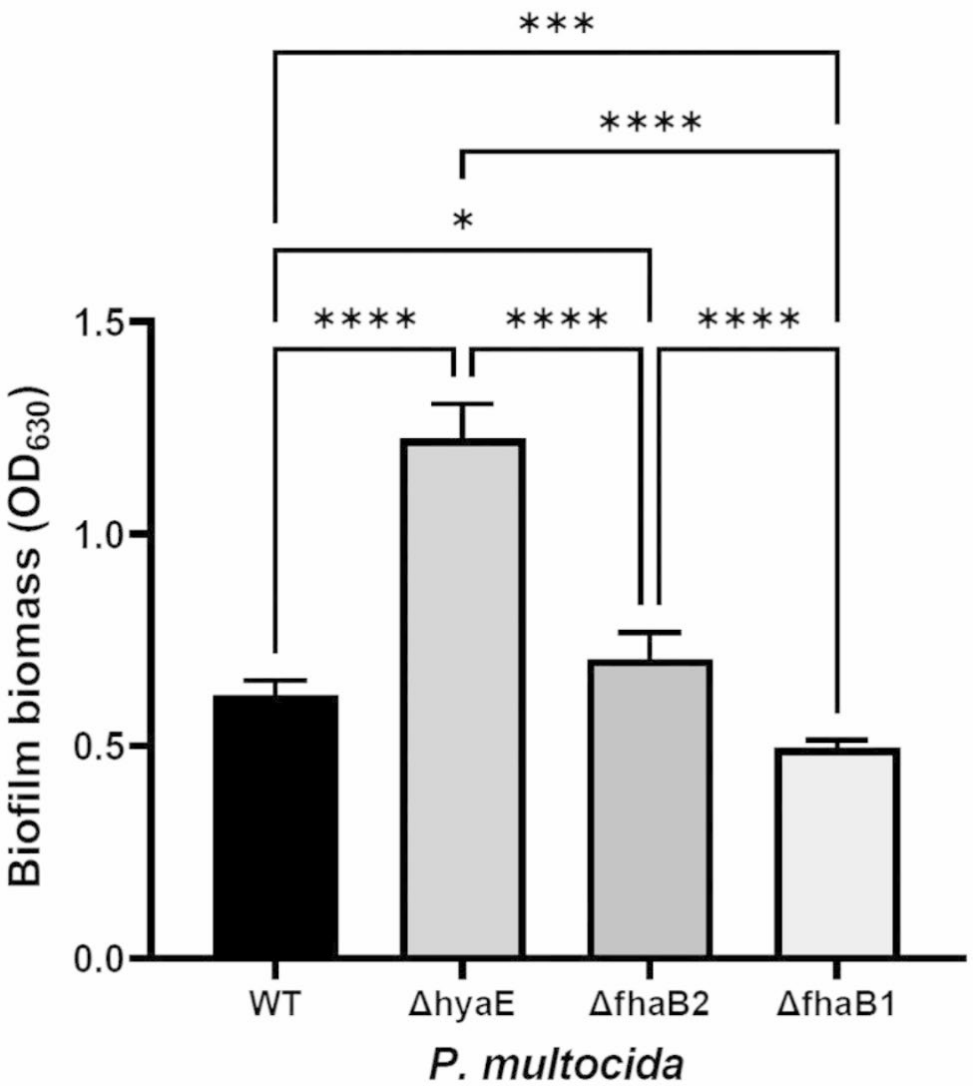
Quantification of *Pasteurella multocida* biofilm biomass. The total biomass of biofilms produced by *P. multocida* wild-type, Δ*hyaE*, Δ*fhaB1*, and Δ*fhaB2* mutant strains grown in colorless RPMI-1640 medium in 24-well plates at 37ºC for 48 h were quantify using crystal violet staining method. The absorbance of crystal violet dissolved in glacial acetic acid was measured spectrophotometrically (OD_630nm_). Bars indicate mean and standard deviation of total biomass of biofilms from three independent experiments performed in four replicate wells for each strain. * *P* = 0.0223; *** *P* = 0.006; ****P < 0.0001

**Table 1:** Oligonucleotides used in this study

Oligonucleotide	Sequence	Target	Product
fhaB1F	5`-CTCAACCAGCGCTTAACTCGC-3`	primer used to generate fhaB1 fragment	fhaB1 bp 131–3003
fhaB1R	5`-GTTTTCTTGTCTAACTGTACACTGTTA-3`	primer used to generate fhaB1 fragment	
fhaB1F	5`-CTCAACCAGCGCTTAACTCGC-3`	forward primer RT PCR analysis set 1	fhaB1 bp 131–317
fhaB1RT1	5`- AATTCAGGTTTTCTT TGTACCACTG-3`	reverse primer RT PCR analysisset 1	
fhaB1F	5`-CTCAACCAGCGCTTAACTCGC-3`	forward primer RT PCR analysis set 2	fhaB1 bp 131–700
fhaB1RT2	5`-CAATGCCATTTTGGTTCGCAA − 3`	reverse primer RT PCR analysisset 2	
fhaB1F	5`-CTCAACCAGCGCTTAACTCGC-3`	forward primer RT PCR analysis set 3	fhaB1 bp 131–926
fhaB1RT3	5`-GTGATATCGGTTTCAGAATTTCCT-3`	reverse primer RT PCR analysisset 3	
Ts ori F	5`-GATCCCTTTTTCTGTAATCT-3`	forward primer Ts origin of replication	Tsorigin1160 bp
Ts ori R	5’-GATCATAGGCTCAATTCTCG-3`	reverse primer Ts origin of replication	
KanR F	5`-ATGAGCCATATTCAACGG-3`	forward primer Tn903 kan resistance element	860 bp
KanR R	5`-TCAGAAAAACTCATCGAGCATC-3`	reverse primer Tn903 kan resistance element	

### Generation of *P. multocida* mutant using a temperature-sensitive replacement plasmid

*P. multocida* P-1059 cells were grown in Columbia broth to an optical density (OD_600nm_) of 0.5 and treated with 250 units of hyaluronidase type 1-S (Sigma Chem. Co. St. Louis, MO) for 10 min on ice to remove hyaluronic acid capsule. The bacteria were pelleted by centrifugation at 5000 × g for 15 min and washed twice with water at 4 °C. Without enzymatic treatment the cells could not be pelleted by centrifugation. The cell pellet was resuspended at a ratio of 1:3 packed bacteria: water and placed on ice. The electroporation competent bacteria (100 μl) were mixed with 1 μl of replacement plasmid DNA mixture in 0.1 cm electroporation cuvette (Bio-Rad Inc, Hercules, CA). Immediately after adding DNA, the bacteria were electroporated (Gene pulser, Bio-Rad) at 18,000 V/cm, 800 ohm, 25 mFd with resultant time constants ranging from 11 to 15 ms. One milliliter of cold Columbia broth was added to the cells, which were recovered at 30 °C for 2 h before being plated onto Columbia agar plates containing 25 μg/ml kanamycin. Approximately 10 colonies containing the Ts replacement plasmid (pTsoriCT109GA189ΔfhaB1Kan^R^) were individually transferred to snap-cap tubes containing 2 ml Columbia broth and 25 μg/ml kanamycin and were incubated overnight at 30 °C. After the growth at 30 °C in broth, a loop of transformed cells was spread onto Columbia agar plates containing 25 μg/ml kanamycin and incubated overnight at 39 °C, the non-permissive temperature for autonomous Ts plasmid replication. Primarily, only single-crossover mutant colonies arose by means of homologous recombination when cells were cultured at high temperature. Randomly selected putative single-crossover mutant colonies were then transferred to broth, without antibiotic selection, and incubated overnight at 30 °C the permissive temperature for autonomous plasmid replication. After three serial passages at low temperature in 2 ml broth cultures, most progeny of the single-crossover mutants were found to be devoid of plasmid. During this step, the active plasmid origin contained on the chromosome caused instability and increased resolution of replacement plasmid from the chromosome resulting in the generation of either wild-type or *fhaB1* mutant progeny.

### Genetic characterization of the *ΔfhaB1* mutant

The putative *P. multocida* Δ*fhaB1*mutant was analyzed by PCR for a deletion within the *fhaB1* gene, for the absence of the Ts origin, TsoriCT109GA189 [23, 24], and the kanamycin resistance element. Single cross-over mutants which contain integrated replacement plasmid, were used as positive control template for the Ts origin and the kanamycin-resistance element. The custom primer pairs were designed and used to amplify *fhaB1* and Ts origin genes are shown in Table 1.

### Quantification of biofilm biomass

The total amount of biofilm biomass produced by *P. multocida* wild-type, Δ*hyaE*, Δ*fhaB1*, and Δ*fhaB2* mutant strains in 24-well plates for 48 h in colorless RPMI-1640 medium was quantify using crystal violet staining method as described previously [25, 26].

### Expression of *fhaB1* in *P. multocida* wild-type and *ΔfhaB1* mutant strains

Real-time PCR analysis was conducted on the wild-type parent (RNA was collected from log and stationary phase cells) and the Δ*fhaB1* mutant (log phase) using the primer pairs shown in Table 1. Briefly, wild-type and Δ*fhaB1* mutant P-1059 were incubated in Columbia broth at 37^°^C with shaking to OD_600nm_ of 0.6, approximately 1 × 10^9^ colony-forming units/ ml (CFU/ml). One ml aliquots were collected, pelleted, and resuspended in RNA protect bacteria (Qiagen, Valencia, CA). The wild-type culture was allowed to enter stationary phase whereupon a second aliquot was collected and stabilized. RNA was extracted from pellets of the three stabilized bacteria preparations using bead disruption in Trizol reagent (ThermoFisher Scientific, Wilmington, DE) and RNeasy (Qiagen) kits. The purified RNA was quantitated and standardized using a Nanodrop spectrophotometer (ThermoFisher Scientific). Reactions were carried out using a SuperScript III protocol in 50 μL total volume containing 25 μL 2 × reaction mix, 0.5 μg RNA, 10 μM each primer, 2 μL SSIII/Taq mix, and 0.5 μL SYBR Green. Cycling was done in a Cepheid Smart Cycler (Sunnyvale, California, USA) with 5 min 50^°^C for first strand synthesis, 20 sec 95^°^C denaturing, and 40 cycles of 1 sec 95^°^C, 20 sec 50^°^C. A final melt temperature analysis was conducted from 60^°^C to 95^°^C at 0.2 C/sec (Table 2). Three primer pairs were used (Table 1), of which set 1 and set 2 were upstream of the genetic deletion and set 3 was downstream.

**Table 2:** Real-time PCR analyses of *fhaB1* transcripts

Primer set	Template RNA	Ct	Tm (°C)
1	P-1059 wild-type log-phase	16.58	80.05
1	P-1059 wild-type stationary	18.07	80.16
1	P-1059 Δ*fhaB1* mutant	17.5	80.34
2	P-1059 wild-type log-phase	18.82	81.27
2	P-1059 wild-type stationary	20.9	81.14
2	P-1059 Δ*fhaB1* mutant	19.73	81.08
3	P-1059 wild-type log-phase	18.58	81.37
3	P-1059 wild-type stationary	19.54	81.61
3	P-1059 Δ*fhaB1* mutant	28.79	63.46

Ct = cycle threshold, Tm = melting temperature

### Evaluation of virulence of the *P. multocida* wild-type parent and *ΔfhaB1* mutant strains in turkey poults

**Fig. 4:**
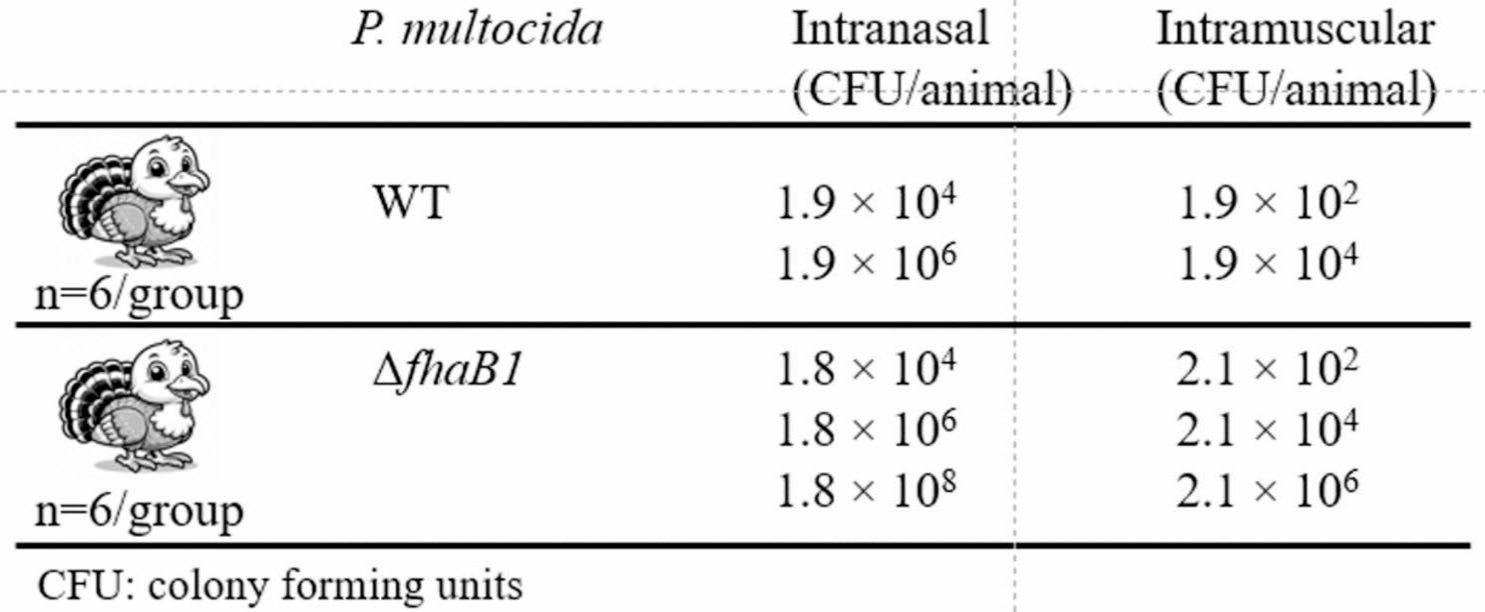
Detailed experimental procedure

All the animals used in this study was housed in an ABSL2 facility and handled as described under ethics statement (Protocol number: ARS-3947). The P-1059 wild-type and Δ*fhaB1* mutant strains (10^8^ CFU) were intravenous injected into turkeys prior to challenge to ensure that the two strains were animal adapted. Pure *P. multocida* cultures were recovered from the liver tissue from deceased birds and stored at -80^°^C. The *P. multocida* strains from single passage liver samples were cultured in Columbia broth to an OD_600nm_ of 0.4 prior to the inoculation of turkey poults. To achieve the intended bacterial numbers (or colony forming units, CFU) for the inoculation, *P. multocida* inocula were centrifuged, and the bacterial pellets were diluted in Gibco™ Earle’s balanced salt solution without phenol red (EBSS, ThermoFisher Scientific). Bacterial numbers in the challenge dilutions were determined by colony plate counts of serial dilutions. Five-week-old turkey poults (Willmar Poultry Co., Willmar, MN) were divided into 10 groups (six birds/group), and were intranasally or intramuscularly (breast muscle) inoculated *P. multocida* wild-type or Δ*fhaB1* mutant (Fig. 4). The birds were observed for signs of disease three times per day for seven days post challenge. Birds showing signs of fowl cholera (ruffled feathers, ataxia, or dehydration) were euthanized by intravenous administration of sodium pentobarbital (Sleep-away, Fort Dodge Animal Health, Fort Dodge, IA). Swab specimens were aseptically collected from livers and tracheas of dead birds then were spread onto a quarter of Columbia blood agar base plates. To isolate single bacterial colonies, three consecutive quadrants were streaked with a sterile loop. To confirm their identity, representative colonies were selected and subjected to PCR amplification using the *fhaB1*primer set (Table 1). At the end of this experiment the remaining birds were euthanized, and liver and trachea tissue samples were processed as described above.

### Statistical analysis

The total biomass of biofilms produced by *P. multocida* wild-type strain, Δ*hyaE*, Δ*fhaB1*, and Δ*fhaB2* mutants were determined spectrophotometrically (OD_630nm_) and presented as mean absorbance with corresponding standard deviation. One-way ANOVA was used to compare biofilm biomass using GraphPad Prism (version 9.5.1., GraphPad Software LLC, Boston, MA). The term significant indicates a value of *P* < 0.05.

## Results

### Construction of *P. multocida ΔfhaB1* mutant

To investigate whether the FhaB1 protein plays any role in fowl cholera, we generated *P. multocida* strain P-1059 (A:3) isogenic Δ*fhaB1* mutant of with a replacement plasmid containing the Ts origin of replication, oriCT109GA189. The Ts replacement plasmid also contained a PCR derived DNA fragment of P-1059 *fhaB1* extending from 131 to 3002 bp in the coding region of the gene (Fig. 2) and a Tn903 kanamycin resistance element. To generate a deletion within the *fhaB1* fragment, treatment with the restriction enzyme *Eco*RV was used to excise bases 853 through 1942 of the gene fragment Fig. 2A and B.

Upon resolution of the Δ*fhaB1* replacement plasmid from the chromosome of single-cross-over mutants, approximately 2% possessed the desired *fhaB1* mutation as determined by PCR analysis, all other clones analyzed had resolved plasmid to generate wild-type colonies. The *P. multocida* Δ*fhaB1* mutants were characterized by PCR with the primer pairs shown in Table 1. PCR products of the expected sizes were produced with the wild-type parent (∼ 3 kb) and mutant strains (∼ 2 kb) using the *fhaB1* primer pair (Fig. 2C, Supplementary Fig. 1). No PCR products were generated when the Δ*fhaB1* strain was used as template with either the Tsori or Kan^R^ primer pairs. The single cross-over mutants, containing replacement plasmid within the chromosome, generated the expected Tsori and Kan^R^ PCR products (data not shown). The Ts origin of replication and the kanamycin resistance element were absent in all kanamycin sensitive colonies analyzed following resolution of the replacement plasmid.

To evaluate the growth rates of *P. multocida* wild-type and mutant strains grown in Columbia broth at 37^°^C, sequential absorbance (OD_600_) readings and viable cell counts were used. As expected, both *P. multocida* wild-type parent and Δ*fhaB1*mutant strains showed similar growth rates when cultured in-vitro (data not shown).

### *P. multocida* Δ *fhaB1* mutant produced less biofilm

Both wild-type and mutant strains produced biofilms, but in different amounts. *P. multocida* capsular mutant (Δ*hyaE*) produced the highest amount of biofilm biomass compared to wild-type and Δ*fhaB* mutant strains (Fig. 3, *P* < 0.0001). Surprisingly, Δ*fhaB1* mutant produced a lower amount of biofilm biomass compared to wild-type strain (Fig. 3, *P* < 0.006). No difference in biofilm biomass amount was observed between wild-type and Δ*fhaB2* mutant strains (Fig. 3, *P* = 0.0223).

### Expression of *fhaB1* by *P. multocida* strain P-1059

One tube real-time reverse transcriptase PCR (RT-PCR) analysis of *P. multocida* RNA was used to probe for *fhaB1* transcripts in wild-type P-1059 and in Δ*fhaB1* mutant strains. The three primer pairs shown in Table 1 generated products using wild-type cells as template, as did the Δ*fhaB1* mutant with the first two primer pairs but not with the third primer pair because the downstream primer was complementary to a region deleted within the *fhaB1* deletion (Table 2). Cycle threshold values (Ct) of *P. multocida* wild-type log phase and stationery, and Δ*fhaB1* mutant strains were very similar among all three primer sets indicating similar *fhaB1* transcripts expression levels (Ct ∼ 16–21). A higher Ct value for Δ*fhaB1* mutant with primer set 3 further suggest the deletion (Ct ∼ 29). These findings confirmed the expression of *fhaB1* transcripts in both wild-type and Δ*fhaB1* mutant strains.

### Virulence of the *P. multocida ΔfhaB1* mutant in turkeys

The inoculation of 1.9 × 10^2^ or 1.9 × 10^4^ CFU (per bird) wild-type parent strain via i.m route resulted in a 100% death in the birds (lethal dose 100; LD100; Fig. 4; Table 3) and all birds died within 24 h with evidence of acute fulminant fowl cholera. Heavy growth of pure *P. multocida* was obtained from the liver, spleen, and nasal specimens. Likewise, all birds receiving i.m. doses of 2.1 × 10^2^, 2.1 × 10^4^, or 2.1 × 10^6^ CFU of the Δ*fhaB1* mutant also died within 24 h with acute fulminant fowl cholera. Bacteria recovered from the tissues of dead animals receiving the mutant were analyzed by PCR which generated ∼ 2 kb product consistent for the Δ*fhaB1* mutant.

**Table 3:** Comparative virulence of *P. multocida* P-1059 wild-type and Δ*fhaB1* mutant strains as determined by intranasal or intramuscular challenge in turkeys

Route	Strain	Dose (CFU)	Deaths/Group		Mean survival (days)
			*Dead	#Euthanized	
Intranasal	WT	1.9 × 10^4^	3/6	0/6	3.1
		1.9 × 10^6^	4/6	1/6	3
	Δ*fhaB1*	1.8 × 10^4^	4/6	0/6	3.2
		1.8 × 10^6^	5/6	1/6	2.8
		1.8 × 10^8^	5/6	1/6	2.8
Intramuscular	WT	1.9 × 10^2^	5/6	1/6	1
		1.9 × 10^4^	5/6	1/6	< 1
	Δ*fhaB1*	2.1 × 10^2^	2/6	4/6	1
		2.1 × 10^4^	5/6	1/6	< 1
		2.1 × 10^6^	6/6	0/6	< 1

WT = wild-type *P. multocida* strain P-1059; *Deaths due to fowl cholera; ^#^Euthanized due to severe signs of fowl cholera

Five of the six birds given the high intranasal dose of wild-type bacteria died (1.9 × 10^6^ CFU), and three of six given the low dose (1.9 × 10^4^) also died within 3 dpi (Table 3; Fig. 4). All birds dosed with the parent strain that survived to the end point of the study were listless and emaciated. Heavy pure growths of wild-type *P. multocida* were obtained from the liver and spleen specimens. Likewise intranasal exposure of the Δ*fhaB1* mutant resulted in death rates and survival times comparable to that of the parent strain (Table 3; Fig. 4). PCR analysis of bacteria recovered from nasal clefts, spleens or livers of this group indicated as expected, that the bacterial growth was *P. multocida fhaB1* mutant.

## Discussion

The *fhaB1* gene of *P. multocida* strain P-1059 comprises an open reading frame of 7,854 bases and is situated upstream of the adjacent *fhaB2* which possesses an open reading frame of 12,807 bases. Each *fhaB* is tightly linked with their respective upstream *fhaC* transporter gene and both show a high degree of similarity to the two-partner secretion systems found in various Gram-negative bacteria (Fig. 1). Genetic comparison of the two exoproteins revealed a shared identity of greater than 50% and both FhaBs possess sequence motifs that are recognized as virulence factors of the IbpA of *Histophilus somni*, the YopTs of *Yersinia* and the FhaBs of *Bordetella* species [27]. Yet unlike results observed with a P-1059 *fhaB2* mutant [16], inactivation of P-1059 *fhaB1* did not reduce virulence in turkeys.

Genomic sequencing showed that the *fhaB1* is present in all avian *P. multocida* sequenced to date [21]. Our RT-PCR analyses confirmed the expression of *fhaB1* transcript in in-vitro cultured bacteria. Whether administered intranasally or intramuscularly, the Δ*fhaB1* mutant of *P. multocida* avian strain P-1059 A:3 remained as virulent to turkeys as the wild-type parent strain in our challenge studies. In contrast to our findings, Guo et al. [28] has reported that an insertional Δ*fhaB1* mutant produced in an avian *P. multocida* strain was attenuated in chickens when administered intranasally and to a lesser degree upon intramuscular delivery. An obvious reason accounting for these divergent results could be due to the different strains used in these studies. In this work, *P. multocida* strain P-1059 serovar A:3 was selected while Guo et al. [24] used the avian *P. multocida* isolate C48-102 which was characterized as capsular serogroup type A. The *P. multocida* Δ*fhaB1* mutant examined here was generated using a temperature-sensitive plasmid which resulted in a “clean” deletion devoid of any foreign DNA. Gene inactivation by Guo et al. [24] was produced through insertional mutagenesis whereby a functional kanamycin resistance element was introduced near the 3` terminus of *fhaB1* gene. The use of antibiotic genes to create insertional mutations within target genes, however, can impact neighboring gene expression and in doing so may obscure the effect(s) on virulence attributed to target gene inactivation. Examples of the influence that active antibiotic resistance elements can exert on neighboring genes include polar termination of distal operon expression [29] and altered regulation of adjacent genes through promotion associated with the insertion marker [30]. Also, turkeys are much more susceptible to fowl cholera than are chickens [8], which, in part, may account for the differences in virulence reported for the two *P. multocida fhaB1* mutants. In this study both the *ΔfhaB1* mutant and the parent used here were passaged through turkeys prior to the pivotal animal comparisons to ensure that virulence was not altered as a consequence of the numerous in-vitro passages required for mutant construction.

Because *P. multocida fhaB1* is present and highly conserved in all *P. multocida* strains sequenced to date, it is tempting to speculate on possible biological roles for this conserved protein: one of which may be involved with biofilm establishment. Filamentous hemagglutinin proteins are known to contribute to bacterial biofilm formations [31, 32] which are matrix-enclosed bacterial assemblages that can adhere to a variety of host sites and cause chronic infections. Biofilm formations are characterized by resistance to innate immune defense mechanisms and to antibiotics [33]. *P. multocida* has been shown to produce biofilms both in vitro and in vivo [22]. Our biofilm study confirmed the production of biofilms by wild-type and *fhaBs* mutant strains, however, Δ*fhaB1* mutant produced the lowest amount of biofilm. Contrary to our observation, *Bordetella pertussis* FHA mutants failed to produce biofilms [32]. Therefore, the observed discrepancy between two FHA mutants, *P. multocida* and *B. pertussis*, is not clear. In vivo, *P. multocida* is often cultured with *H. somni* biofilm obtained from heart and lung samples of infected cattle [34]. Widely distributed localized infections may follow the acute septicemic form of fowl cholera suggesting that *P. multocida* may be residing as biofilms in chronically infected birds. Due to the challenge methods and the highly sensitive animal model used in this study, upon entering the bloodstream the *P. multocida fhaB1* mutant, as well as the wild-type parent, are likely to have remained in the highly virulent planktonic form which engendered the acute form of fowl cholera that was rapidly lethal in turkeys. Since we did not study surface adhesion properties of *fhaB1* mutant in respiratory epithelial cells of turkey, whether *fhaB1* mutant alter the gene regulation networks, or interactions with other virulence factors is currently unknown. Future experiments are planned to investigate whether the *P. multocida* FhaB1 protein plays a role establishment of biofilm formation and chronic infection in poultry.

## Conclusions

Unlike a previous study in turkeys with avian *P. multocida ΔfhaB2* P-1059 mutant, inactivation of *fhaB1* P-1059 did not reduce virulence in either intranasal or intramuscular challenge routes. These findings suggest that the large and highly conserved FhaB1 protein is not necessary for the development of acute fowl cholera in turkeys.

## Electronic supplementary material

Below is the link to the electronic supplementary material.

Supplementary Material 1

## Data Availability

No datasets were generated or analysed during the current study.

## Abbreviations

ABSL: Animal biosafety level
ANOVA: Analysis of variance
CFU: Colony forming units
DNA: Deoxyribonucleic acid
Fha: Filamentous hemagglutinin
Hya: Hyaluronic acid biosynthesis
I.M: Intramuscular
mRNA: Messenger ribonucleic acid
OD: Optical density
Omp: Outer membrane proteins
RT-PCR: Real-time polymerase chain reaction assay
S.C: Subcutaneous
