# Supplementary material for: Pasteurella multocida filamentous hemagglutinin B1 (fhaB1) gene is not involved with avian fowl cholera pathogenesis in turkey poults
Source: BMC Vet Res. 2025 Mar 26;21:207. doi: 10.1186/s12917-025-04668-1 (PMC11938644; doi:10.1186/s12917-025-04668-1)

**Supplementary information**

***Pasteurella multocida* filamentous hemagglutinin B1 (*fhaB1*) gene is not involved with avian fowl cholera pathogenesis in turkey poults.**

Rohana P. Dassanayake^1^, Robert E. Briggs^1^, Bryan S. Kaplan^1^, Harish Menghwar^1,2^, Carly Kanipe^3^, Eduardo Casas^1^, Fred M. Tatum^1*^

^1^Ruminant Diseases and Immunology Research Unit, United States Department of Agriculture, Agricultural Research Service, National Animal Disease Center, Ames, IA 50010, USA.

^2^Oak Ridge Institute for Science and Education (ORISE), ARS Research Participation Program, Oak Ridge, TN 37830, USA.

^3^Infectious Bacterial Diseases Research Unit, United States Department of Agriculture, Agricultural Research Service, National Animal Disease Center, Ames, IA 50010, USA.

**Figure S1**

***Pasteurella multocida* avian strain P-1059 filamentous hemagglutinin B1 (*fhaB1*) gene PCR products.** The wild-type parent (W) and Δ*fhaB1* mutant (M) strains of *P. multocida* were analyzed by PCR using the primer pair fhaB1F (5`-CTCAACCAGCGCTTAACTCGC-3`) and fhaB1R (5`-GTTTTCTTGTCTAACTGTACACTGTTA-3`). PCR product sizes: wild-type parent (~3 kb) and mutant strains (~2 kb).

MW W M

(kb)


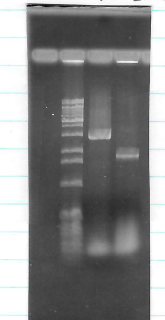

Supplement: Supplementary file 1 — Supplementary Material 1 [file 12917_2025_4668_MOESM1_ESM.docx]
